# Spiritual Dryness as a Measure of a Specific Spiritual Crisis in Catholic Priests: Associations with Symptoms of Burnout and Distress

**DOI:** 10.1155/2013/246797

**Published:** 2013-06-17

**Authors:** Arndt Büssing, Andreas Günther, Klaus Baumann, Eckhard Frick, Christoph Jacobs

**Affiliations:** ^1^Quality of Life, Spirituality and Coping, Institute for Integrative Medicine, Faculty of Health, Witten/Herdecke University, Gerhard-Kienle-Weg 4, 58239 Herdecke, Germany; ^2^External Senior Research Fellow Freiburg Institute for Advanced Studies (FRIAS), University of Freiburg, 79104 Freiburg, Germany; ^3^Pastoral Psychology and Sociology, Faculty of Theology Paderborn, Kamp 6, 33098 Paderborn, Germany; ^4^Caritas Science and Christian Social Work, Faculty of Theology, Albert-Ludwigs University, Platz der Universität 3, 79098 Freiburg, Germany; ^5^Internal Senior Research Fellow Freiburg Institute for Advanced Studies (FRIAS), University of Freiburg, 79104 Freiburg, Germany; ^6^Munich School of Philosophy & Professorship of Spiritual Care, University of Munich, Marchioninistraße 15, 81377 Munich, Germany

## Abstract

Spirituality/religiosity is recognized as a resource to cope with burdening life events and chronic illness. However, less is known about the consequences of the lack of positive spiritual feelings. Spiritual dryness in clergy has been described as spiritual lethargy, a lack of vibrant spiritual encounter with God, and an absence of spiritual resources, such as spiritual renewal practices. To operationalize experiences of “spiritual dryness” in terms of a specific spiritual crisis, we have developed the “spiritual dryness scale” (SDS). Here, we describe the validation of the instrument which was applied among other standardized questionnaires in a sample of 425 Catholic priests who professionally care for the spiritual sake of others. Feelings of “spiritual dryness” were experienced occasionally by up to 40%, often or even regularly by up to 13%. These experiences can explain 44% of variance in daily spiritual experiences, 30% in depressive symptoms, 22% in perceived stress, 20% in emotional exhaustion, 19% in work engagement, and 21% of variance of ascribed importance of religious activity. The SDS-5 can be used as a specific measure of spiritual crisis with good reliability and validity in further studies.

## 1. Introduction

Religion and spirituality have become a matter of empirical research in various contexts since the last 30 years. Various operationalizations have been proposed considering various aspects of spirituality [1, 2]. There is an increasing number of studies indicating relations between spirituality/religiosity and health (reviewed by [3]). Although results are not always consistent (reviewed by [4]) and often dependant on specific populations, cultures, and specific measures [5], many studies found positive associations between specific facets of spirituality and psychological well-being (reviewed by [6, 7]), quality of life (reviewed by [8]), and coping (reviewed by [9]). A systematic review on the “potential beneficial or harmful effects of religious/spiritual coping” indicated that this specific form of coping may be beneficial to “maintaining self-esteem, providing a sense of meaning and purpose, giving emotional comfort and providing a sense of hope” [10]. 

However, less is known about the consequences of the lack of positive spiritual feelings, although it is an often reported phenomenon. Those who care for others, either as health care professionals or spiritual advisors and priests, may experience phases of crisis which can be due to external factors, that is, work overload, structural changes in the work processes, conflict with colleagues, low credit by superiors, and so forth, and also due to internal factors, that is, psychological traits and capacities, own resources to rely on, and so forth. These factors may have an impact on professional “functioning,” resulting either in positive work engagement and dedication on the one hand [11], or burnout and reduced life satisfaction on the other hand [12]. As a consequence it is to expect that spiritual life and fulfilment will be affected too either in a positive or negative way. In fact, first findings among protestant pastors show that higher spirituality was related to healthier work-related behaviour patterns [13]. But what about spiritual feelings of those who professionally care for the spiritual side of others, what about chaplains and priests who are suggested to have an active spiritual life?

Although controversially discussed, Mother Teresa (Agnes Gonxha Bojaxhiu, 1910–1997), one of the blessed of Catholic Church, is recognized as a very sacrificial and devoted nun working for the sick in the slums of Calcutta (India). Receiving the Nobel Peace Prize for her work in 1979, she inspired several, both lay persons and ordained, to minister to the poor, sick, and dying all over the world. However, after she has passed away, and during the process of beatification, the world started to recognize her statements of spiritual “dryness,” “darkness,” and “loneliness” [14]. In September 1979, she said to Rev. Michael Van Der Peet: “Jesus has a very special love for you. As for me, the silence and the emptiness is so great that I look and do not see, listen and do not hear” [14]. This and other statements indicate long-lasting phases (or even states) of spiritual crisis even in those who are suggested to be filled with strong faith. These phases can be either a complete detachment (in terms of loss of faith) or part of spiritual growth as described as “dark night of the soul” by the Spanish 16th century mystic and Carmelite friar John of the Cross (San Juan de la Cruz, 1542–1591). Another Spanish 16th century mystic, St. Ignatius of Loyola, suggested practical rules for discernment of spirits, aiming at understanding times of consolation and desolation in a human being's life trajectory. Ignatius describes desolation as “darkness of soul, disturbance in it, movement to things low and earthly, the unquiet of different agitations and temptations, moving to want of confidence, without hope, without love, when one finds oneself all lazy, tepid, sad, and as if separated from his Creator and Lord” (Spiritual Exercises no. 317). 

So far no research has been done to study systematically the lack of positive spiritual feelings. Yet many spiritual men and women refer to such feelings as a form of absence of God and a lack of spiritual comfort as illustrated by the example of Mother Theresa. Thus, we intended to establish a psychological construct that seizes these feelings of spiritual bereavement and dryness and analysed their relations with personal characteristics, situational factors, and effects for psychological health and to analyse predictors of such specific feelings. 

## 2. Hypothesis

We hypothesized that the feelings of spiritual dryness and bereavement are associated with distress, depressive symptoms and burnout, decreased work engagement, less self-rated psychological health, and finally reduced overall life satisfaction. Personal characteristics such as pessimism, low sense of coherence, and low self-efficacy as well as occupational factors as work burden, missing appreciation by superiors, low autonomy, and work stress are supposed to lead to an impairment of positive spiritual feelings and thus can aggravate phases of spiritual crisis or even the sensation of abandonment by God. Further, for the sake of construct validity, spiritual dryness should be negatively associated to positive spiritual experiences, the self-ascribed importance, and amount of spiritual practices.

Thus, the aim of this study is to demonstrate first results for the validation of the new construct “spiritual dryness” and present empirical evidence of the expected associations.

## 3. Methods

### 3.1. Participants

All individuals of this anonymously conducted cross-sectional study were informed about the purpose of the study, were assured of confidentiality and their right to withdraw at any time, and asked to provide informed consent. They were recruited among Catholic priests of a huge German diocese (i.e., Paderborn).

The priests were informed about the study by the personnel manager of the dioceses and invited by a separate letter from the authors to participate in the study. Participation was possible by pencil and paper version or online questionnaire.

425 out of 998 persons compiled the questionnaire, which means a participation rate of 43%. Among the respondents, 297 answered the print version (70%), and 128 preferred the online form (30%). Elderly priests favoured the print version. Most worked as parish priests (*n* = 241; 124 with leadership function, 86 with cooperative function, 31 as chaplains), 30 in the field of pastoral counselling, 116 were already retired (out of whom 84 still assisted in a parish), and 29 had other duties and responsibilities (academic and management). The priests' mean age was 58 years. The distributions of age and occupational tasks were representative for the diocese.

### 3.2. Measures

#### 3.2.1. Spiritual Dryness Scale

We intended to operationalize and make measurable feelings of “spiritual dryness.” These phases of spiritual crisis are in most cases transient, and thus we asked for the general experience rather than for acute phases of putative burnout and/or depression. We assume that these feelings can be aggravated by work burden, personal traits, and lack of own spiritual engagement.

The intended instrument consists of two parts. First, items to measure whether or not individuals already have experienced such phases of “spiritual dryness”, feelings that God is distant, that one's prayers go unanswered, to be “spiritually empty” or not being able to give any more (both in terms of a spiritual exhaustion), and finally feelings to be abandoned by God. The second part of the instrument addresses reactions towards these experiences, that is, whether one has found ways to deal with these feelings (the individual strategies can be added as free texts), whether these feelings inspire one all the more to help others, and whether or not individuals have experienced greater spiritual serenity and depth after these phases.

The specific items refer to statements found in the testimony of Mother Teresa's experiences of spiritual “dryness,” “darkness,” and “loneliness” [14]. The items of this instrument were formulated in such a way that they fit to daily life experiences of religious individuals, either lays or ordained. Response options were “not at all” (1), “rarely” (2), “occasionally” (3), “fairly often” (4), and “regularly” (5). 

#### 3.2.2. Burnout

To measure burnout, we used the Maslach Burnout Inventory (MBI) which has three subscales [15]. The emotional exhaustion subscale (*α* = .90) measures feelings of emotional overextension and exhaustion by one's work; the depersonalization subscale (*α* = .79) measures an “unfeeling and impersonal response” towards recipients; while the personal accomplishment subscale (*α* = .71) measures feelings of competence and successful achievement in one's work with others.

Specific items are, for example, “I feel emotionally drained from my work,” “Working with people all day is really a strain for me,” “I feel used up at the end of the workday,” “Working with people directly puts too much stress on me”, and so forth. All items are scored on a 7-point scale ranging from (experienced) “never” to “every day.” 

#### 3.2.3. Psychological Distress

To measure psychological distress, Derogatis [16] developed the 18-item brief symptom inventory (BSI-18), a short form of the symptom check list (SCL-90-R). This instrument has three scales with 6 items each, that is, somatization, depression and anxiety.

Specific items are feelings of worthlessness, loneliness, and being down, no interest in things, hopelessness about future, pains in heart and chest, nausea or upset stomach, nervousness, restlessness, scared for no reason, spells of terror or panic.

The German version has good reliability coefficients for the respective subscales (i.e., somatization: *α* = .79; depression: *α* = .84; anxiety: *α* = .84) [17]. All perceptions are scored on a 5-point Likert scale ranging from “not at all” to “very strong.” 

#### 3.2.4. Perceived Stress Scale

The perceived stress scale (PSS) is a 10-item questionnaire to measure the self-perceived stress level in specific situations during the last month [18]. Internal reliability of the original PSS-10 was moderate (*α* = .78) [18]. In our sample reliability was good (*α* = .87).

Specific items are been upset because of something that happened unexpectedly, felt unable to control the important things in life, felt confident about ability to handle personal problems, been angered because of things that happened that were outside of control, could not cope with all the things that one had to do, and so forth.

All items refer to emotions and thoughts and how often one may have felt or thought a certain way. The scores range from 1 (never) to 4 (very often); higher scores would thus indicate greater stress.

#### 3.2.5. Life Orientation

To measure optimistic and pessimistic attitudes, we used the 10-item revised life orientation test (LOT-R) [19]. Internal consistency of the respective subscales with 3 items each (and 4 filling items) is rather weak, that is, optimism: *α* = .69; pessimism: *α* = .68). 

Representative items are “In uncertain times, I usually expect the best,” “If something can go wrong for me, it will,” “I'm always optimistic about my future,” “I rarely count on good things happening to me.” All items are scored on a 5-point scale ranging from “agree a lot” to “disagree a lot.” 

#### 3.2.6. Sense of Coherence

The sense of coherence scale (SOC) is widely used to assess internal strengths of an individual referring to Antonovsky's “salutogentic orientation” [20]. According to theory, this sense of coherence may determine a person's coping with stressors in life. 

Antonovsky primarily developed a 29-item instrument with a putatively one-dimensional structure [20]. For this study, we used the 13-item version of the SOC with 7-point semantic differential; these scales intend to measure comprehensibility (5 items), manageability (4 items), and meaningfulness (4 items). However, there is currently a debate about the factorial structure of the instrument which is highly inconsistent depending on the tested samples. Jakobsson [21] recently tested the construct validity of the 13-item version and reported that the “instrument failed to show acceptable construct validity in any of the tests or in any age group” and that “factor analyses did not support the factor structure proposed by Antonovsky.” Thus, for this analysis we will refer only to the SOC-13 sum score.

Representative items are “Do you have the feeling that you really do not care about what is going on around you?,” “Has it happened that people whom you counted on disappointed you?,” “Until now your life has had: no clear goals—very clear goals and purpose,” “Do you have the feeling that you are in an unfamiliar situation and do not know what to do?,” “Does it happen that you experience feelings that you would rather not have to endure?,” “How often do you have the feeling that there is little meaning in the things you do in your daily life?,” and so forth.

#### 3.2.7. General Self-Efficacy

To assess individuals' self-efficacy, we used the german language general self-efficacy scale (GSE) [22]. The GSE scale has a good to very good internal consistence, that is, Cronbach's alpha in German samples ranging from .80 to .90 [23].

Specific items are “If someone opposes me, I can find means and ways to get what I want,” “When I am confronted with a problem, I can usually find several solutions,” “I am confident that I could deal efficiently with unexpected events,” “No matter what comes my way, I am usually able to handle it.” The 10 items are answered on a 4-point Likert scale ranging from disagreement to agreement. High scores indicate higher (optimistic) self-efficacy.

#### 3.2.8. Work Engagement

The utrecht work engagement scale (UWES) measures “a positive, fulfilling, work-related state of mind that is characterized by vigor, dedication, and absorption” [24]. For this study, we used the 9-item shortened version (UWES-9; alpha ranging between .85 and .92) which has similar psychometric properties than the long version. It has been shown that work engagement is negatively associated with burnout [25] and positively related to work and life satisfaction and self-rated health [24].

Specific items are “I am enthusiastic about my job,” “At my work, I feel bursting with energy,” “At my job, I feel strong and vigorous,” “I am proud of the work that I do,” “I am immersed in my work,” and so forth. The items are scored on a 7-point Likert scale, ranging from “never” to “always/every day.”

#### 3.2.9. Satisfaction with Life

To measure life satisfaction we relied on the German version of Diener's satisfaction with life scale (SWLS) [26]. This 5-item scale (*α* = .92) uses general phrasings such as “In most ways my life is close to my ideal,” “The conditions of my life are excellent,” “I am satisfied with my life,” “So far I have gotten the important things I want in my life,” and “If I could live my life over, I would change almost nothing” [26]. 

The extend of respondents' agreement or disagreement is indicated on a 7-point Likert scale, ranging from “strongly agree” to “strongly disagree.” 

#### 3.2.10. Daily Spiritual Experiences

The instrument was developed as a measure of a person's perception of the transcendent in daily life, and thus the items measure experience rather than particular beliefs or behaviors [27, 28]. Here we used the 6-item version of the daily spiritual experience scale (DSES; *α* = .91) which uses specific items such as feel God's presence, God's love, desire to be closer to God (union), find strength/comfort in God, and touched by beauty of creation [27].

The response categories are “many times a day,” “every day,” “most days,” “some days,” “once in a while” and “never/almost never.”

#### 3.2.11. Importance of Specific Spiritual Practices/Activities

To differentiate various forms of specific spiritual practices, we used the SpREUK-P questionnaire [29, 30]. The generic instrument was designed to measure the engagement in organized and private religious, spiritual, existential, and philosophical practices. In its 24-item version it differentiates 5 factors: (1) religious practices (*α* = .84; i.e., praying, church attendance, religious events, religious symbols, etc.); (2) existential practices (*α* = .83; i.e., self-realization, spiritual development, meaning in life, etc.); (3) humanistic practices (*α* = .76; i.e., help others, consider their needs, do good, connectedness, etc.); (4) spiritual (mind body) practices (*α* = .80; i.e., meditation, rituals, reading spiritual/religious books, etc.); and (5) gratitude/reverence (*α* = .76; i.e., feeling of gratitude, reverence, experience beauty in nature).

With respect to the specific sample, we adjusted some items; that is, we used the term “Holy Communion” instead of “church attendance”, performance of distinct rituals from “other religious traditions”, differentiated meditation as “in the style of Buddhist traditions” or “Christian style;” moreover, we added one item specific for Catholic priests, that is, “liturgy of hours.”

This specific version of the instrument used here asks for the *importance *of these activities (SpREUK-P Ipt) and scores the responses as “not at all,” “somewhat,” “very,” and “indispensable.” These scores referred to a 100% level (“indispensable” = 100%; transformed scale score), which reflects the degree of ascribed importance of the respective practices/activities in their life.

Within this sample of Catholic priests, the 20 items of the SpREUK-P Ipt have a good internal consistency (*α* = .84) and make up 5-6 factors which are consistent with the primary scales: (1) religious practices with two sub-scales, that is, active religious practices (*α* = .78) and passive religious practices (*α* = .56); (2) existential practices (*α* = .75); (3) humanistic practices (*α* = .78); (4) eastern forms of spiritual practices (*α* = .62); (5) gratitude/reverence (*α* = .83). Particularly the 2-item subscale passive religious practices has a weak internal reliability; these two items are observed so far only in this distinct population and should thus not be overestimated.

### 3.3. Statistical Analyses

Descriptive statistics, internal consistency (Cronbach's coefficient *α*), and factor analyses (principal component analysis using Varimax rotation with Kaiser's normalization), as well as analyses of variance, first order correlations, and regression analyses were computed with SPSS 20.0. Structural equation modelling was accomplished with SPSS Amos 20.0.

Given the exploratory character of this study, significance level was set at *P* < .05.

With respect to classifying the strength of the observed correlations, we regarded *r* > .5 as a strong correlation, an *r* between .3 and .5 as a moderate correlation, an *r* between .2 and .3 as a weak correlation, and *r* < .2 as no or a negligible correlation.

## 4. Results

### 4.1. Participants

All priests had a high school education; the majority were between 40 to 60 years of age (Table 1). Most were living alone, only 6.4% in a fraternity. Further characteristics are given in Table 1.

The overall psychological distress (BSI) of the priests must be regarded as elevated (mean = .53, SD = .52; population mean = .22 [31]), while burnout values were in the range of the normal. About 10% of the sample exceeded cut-offs in all three scales of emotional exhaustion, depersonalisation, and reduced personal fulfilment and therefore has to be considered to be compromised by burnout. In confront to the normal population, the sample showed slightly decreased values of sense of coherence (SOC) and considerably lower values of general self-efficacy (GSE). On the contrary, self-reported social support was very high. Work engagement has to be considered on average. The overall life satisfaction was markedly higher (mean = 7.5) than mean values of the population of North-Rhine-Westphalia where the study was performed (mean = 7.0 [32]).

### 4.2. Response Rates to the Respective Experiences of Spiritual Dryness

As shown in Table 2, feelings of spiritual dryness or spiritual emptiness are experienced occasionally by up to 40%, often or even regularly by up to 13%, while the explicit feelings that God is distant or to be abandoned by God were experienced often only by 4–7%, and not at all by 35% and 53%, respectively. This means that spiritual dryness may occur occasionally as a phase of spiritual crisis.

About 60% responded to the items that address concrete actions when these phases were experienced (Table 2). Referring to these respondents, 57% stated that they have found ways to deal with these feelings and 15% rarely or not at all (9% of the whole sample). These feelings inspired 28% of the respondents all the more to help others, while for 37% these feelings were not transformed into concrete actions to help others (22% of the whole sample stated “rarely” or “not at all”); in 35% this reaction occurred occasionally. Greater spiritual serenity and depth were experienced by 33% of the respondents, while 25% did not; in 42% this experience occurred occasionally.

### 4.3. Reliability and Factor Analysis of the Spiritual Dryness Scale

The first 6 items dealing with the concrete experience of these phases of spiritual dryness can be condensed to a *spiritual dryness scale* (SDS) which showed good internal consistency (Cronbach's alpha = .87) (Table 3). The item difficulty (1.31[mean value]/4) was .33; with the exception of the item addressing feelings to be abandoned by God (indicating a bottom effect due to the lack of regular experience), all values were in the acceptable range from .20 to .80.

Factor analysis revealed a Kaiser-Meyer-Olkin value of .84, which as a measure for the degree of common variance indicates its suitability for statistical investigation by means of principal component factor analysis. Exploratory factor analysis pointed to one main factor (eigenvalue = 3.6) which accounted for 60.2% of variance (Table 2).

**Figure 1:**
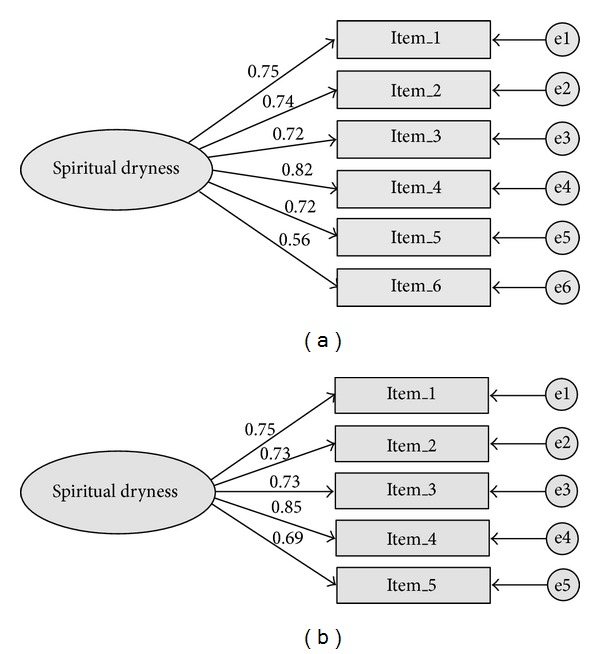
(a) SEM of SDS-6 with standardized factor loadings. (b) SEM of SDS-5 with standardized factor loadings.

Structural equation modelling proved a good fit of a unidimensional solution with model fit characteristics of *χ* ^2^ [df = 9, *N* = 425] = 96.918, *P* < .001, CFI = .92, AIC = 120.918, and SRMR = .049 (Figure 1(a)). The lowest factor loading was observed for item 6 (“I know the feeling of not being able to give any more”) with *r* = .56, while the other 5 items ranged from *r* = .72 to .84. This might be due to the wording of the item with no explicit regard to God or spiritual life. Thus model fit could be improved omitting item 6. The unidimensional 5-item scale proved a very good fit with fit characteristics of *χ* ^2^ [df = 5, *N* = 425] = 48.133, *P* < .001, CFI = .96, AIC = 68.133, and SRMR = .040 (Figure 1(b)). So SEM recommends to omit item 6.

For all further analyses we will thus refer to the 5-item version of the SDS (SDS-5) which lacks item 6. 

### 4.4. Correlation and Regression Analyses

To assess convergent validity of the SDS-5, we performed correlation analyses. As can be expected from a theoretical point of view, the SDS-5 correlated strongly with depressive symptoms and moderately with burnout (MBI), perceived stress (PSS), and pessimism (LOT-R) on the one hand, and strongly negative with daily spiritual experiences (DSEs), moderately negative with sense of coherence (SOC), life satisfaction (SWLS), self-efficacy (GSE), optimism (LOT-R), work engagement (UWES), and with active religious practices (SpREUK-P) and gratitude/awe (SpREUK-P) on the other hand (Table 4). In contrast, there were no significant associations with hours of work, size of the parish/pastoral unit or team size (Table 4); moreover, there was no significant association with age (data not shown).

With respect to criterion validity, we performed regression analyses to assess the variance of health related variables on the one hand, and spiritual experience on the other hand, which can be attributed to the experience of spiritual dryness. As shown in Table 5, the experience of spiritual dryness can explain variance in mental health affections, work engagement, and spiritual activities, that is, 44% of variance in daily spiritual experiences (DSEs), 30% of variance in depressive symptoms, 22% of variance in perceived stress (PSS), 20% of variance in emotional exhaustion (MBI), 19% of variance in work engagement (UWES), and 21% of the importance of active religious activity.

### 4.5. Predictors of “Spiritual Dryness”

Because several variables were empirically observed which might have an impact on the experience of spiritual dryness, we performed multiple regression analyses (stepwise exclusion). The strongest predictor of spiritual dryness was low daily spiritual experiences, followed by depressive symptoms and perceived stress, and as a further negatively associated predictor the importance of active religious activities (Table 6). These variables explain 55% of SDS variance.

As the regression coefficients may be compromised by collinearity, we checked the variance inflation factor (VIF) as an indicator for collinearity. A VIF higher than 10 is indicative for high collinearity. Results suggested that collinearity was not a problem in the respective models. However, in several cases the VIF values ranged up to 1.7 indicating very low but tolerable collinearity in the data.

### 4.6. Mean SDS Scores within the Sample

**Figure 2:**
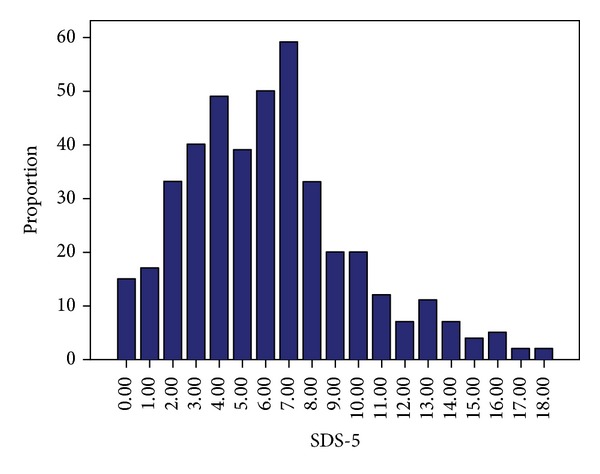
Distribution of SDS scores within the sample (range 0–20).

The SDS-5 sum scores (range 0–20) showed a left skewed Gaussian distribution (Figure 2). The mean score was 6.2 ± 3.7; the 33% percentile was at 4.0 and the 67% percentile at 7.0.

As shown in Table 7, the SDS-5 score of individuals with high emotional exhaustion differs significantly from those with low scores (*F* = 48.6; *P* < .0001). Similarly, individuals with perceived stress have significantly higher scores than those without (*F* = 35.5; *P* < .0001). In contrast, individuals with low daily spiritual activities had significantly higher SDS scores than those with moderate or high activity scores (*F* = 111.5; *P* < .0001). Thus, so far one could assume SDS-5 scores <5 as unremarkable, scores between 5 and 9 as moderate, and scores ≥10 as high. Following this preliminary categorization, 36% of the tested priests were unsuspicious of spiritual crisis, 47% had moderate SDS scores, and 16% high scores.

## 5. Discussion

It was our intention to operationalize feelings of spiritual dryness as a specific measure of spiritual crisis. The resulting 5-item instrument, the SDS-5 scale, has sound psychometric properties, and the findings are congruent with the underlying hypotheses. In fact, we were able to show that “spiritual dryness” is strongly negatively associated with engagement in spiritual activities on the one hand, and positively with variables of distress and character traits on the other hand. Yet there were no significant associations with duration of work during the week, with size of parish/pastoral unit or team size, instead negatively with fulfilling work engagement. Thus, so far we have hints about which variables may contribute or facilitate such phases of spiritual crisis, which finally will impact life satisfaction. A survey of male Anglican priests showed that theological orientations such as liberal versus conservative (“churchmanship”) were strongly associated with clergy satisfaction [33]. US American Catholic priests' vocational satisfaction was found to comprise three factors: External manifestations (e.g., preaching, teaching), internal manifestations (e.g., prayer life, affirmation of God's call), and social manifestations (e.g., relationships with parishioners, appreciation from others) [34].

Although high SDS-5 scores were associated with depressive symptoms and burnout symptoms, they are not necessarily an indicator of affected mental health, as they can simply reflect a transient spiritual crisis one has to deal with. Future analyses have to clarify how long such phases may last (this will also include analyses of test-retest reliability) and which individuals need support to cope with these phases. Our data indicate that in Catholic priests, low spiritual activities and low importance of active religious activities can be predictors of such spiritual dryness. However, it is currently unclear whether reduced spiritual activity is the result of such spiritual crises or one of the conditions. Nevertheless, one cannot ignore that these phases go hand in hand with emotional exhaustion [35], depressive symptoms, and perceived stress; yet, most seem to find strategies to deal with these phases. In fact, 60% of the tested individuals responded to the second part of the questionnaire and thus stated that they already have experienced such phases of spiritual dryness. Among them, 9% stated that they did not find ways to deal with these feelings, while 33% did fairly often or even regularly. Moreover, several priests were inspired by these phases all the more to help others (22%), or these phases finally resulted in greater spiritual serenity and depth (20%), indicating a spiritual transformation of such experiences.

Spiritual traditions of all major religions understand spiritual life and transformation as a journey which is not easy, smooth, and automatic but a pilgrimage of self-transcendence implying temptations and fatigue, struggles, and discouragements. This is also true for the Christian tradition: self-transcendence inherent to love of God and neighbour is challenging the person both in her or his prayer life, relationship to self and others, as well as work or ministry. Love of God and love of neighbour are understood as belonging together in intimate union and interrelation. Due to the human condition of self-transcendence which implies decisions and renunciations with their intrinsic tensions, such union and interrelation is not taken for granted, but rather an object of illusions about the dynamic meanings of joy and fatigue: joy may show truly or only apparently what is good and true, and fatigue may show truly or only apparently that one is on the wrong track. So, on has to state progressive and regressive spiritual processes which lead ahead or astray on the journey of faith.

For Catholic priests, who professionally care for the spiritual sake of others, their prayer life as well as ministry may be experienced as sources of joy and trust which energize the personal and communitarian spiritual journey. Prayer life and ministry may also become annoying or boring, aloof, and even tedious for the priests at some points or periods of their spiritual journey. Such experiences may be regarded as necessary parts of perseverance on the journey and inspire their empathy and solidarity with persons in spiritual turmoil; they may also, in an opposite way, reduce their readiness for ministry and even lead to leaving their vocation [36]. 

So far we have to state that Catholic priests do know the experience of “helpless helper,” when their own spiritual activities ironically seem to be fruitless, resulting in phases of burnout and distress [35, 37]. Then the required confidence in their own spiritual resources needs to be rediscovered and reassured. Which strategies were used by the individuals investigated herein remain to be analysed in the next steps of the study. 

Further research should investigate the causal relationship of personal, spiritual, and health interconnections and carve out more clearly the causal antecedents and effects of spiritual dryness. Also the transitory character of spiritual dryness needs further attention. Which circumstances and personal characteristics contribute to overcome a spiritual crisis and which lead to an aggravation and perpetuation of this state of crisis? Are there any characteristics of the person or the environment which may predict the positive or negative development of a spiritual crisis?

Interconnected with this question, the stability of feelings of spiritual crisis should be inquired. As for many religious people this is a transient state of mind; even very gifted religious man and women report this as a very stable experience that lasted for many years of their lives. Narratives of religious people witness that these experiences led negatively either to an increasing desolation with even depressive symptoms and in a final step to the loss of faith in God or positively to various efforts to overcome these negative feelings often ensued by a deepened sense of peace, consolation, and closeness to God. The “night of faith” is to be understood as an ambivalent phenomenon leading to spiritual growth or the alienation from God [38]. Thus, it might be hypothesized that short phases of spiritual crisis may have even positive effects of purification and deeper faith, while long-lasting phases of spiritual dryness have negative consequences for the individual resulting in hopelessness and spiritual distress.

## 6. Conclusion

Compared to the measures of depressive symptoms, burnout, and perceived stress, the SDS measures a different construct. So far, data indicate adequate reliability and validity; moreover, the predictors of spiritual dryness are sound from a theoretical point of view, and thus the instrument can be used as a specific measure of spiritual crisis in specific populations; these studies are currently under way. It is so far striking that this specific spiritual crisis is experienced by a relatively large number of priests which in most cases did find strategies to deal with such crisis. 

## Figures and Tables

**Table 1:** Characteristics of 425 Catholic priests.

Variables	%*
Age categories (born between) (%)	
1980 and 1989	3
1970 and 1979	14
1960 and 1969	28
1950 and 1959	16
1940 and 1949	11
1930 and 1939	22
1920 and 1929	7
Living situation (%)	
Alone	40.2
Alone but with external housekeeper	28.2
With housekeeper	16.6
With other priests	6.5
With others (i.e., family)	3.4
Other living facilities	5.1
Duration of work (%)	
<10 h per week	5
10–20 h per week	11
20–30 h per week	7
30–40 h per week	9
40–50 h per week	26
50–60 h per week	28
60–70 h per week	12
Work engagement (UWES) (mean ± SD, range)	4.1 ± 1.1 (1–7)
Health associated and personal variables (mean ± SD, range)	
Distress (BSI)—general severity index	9.5 ± 9.5 (0–58)
Body mass index (BMI) (normal range)	27.4 ± 4.5 [20–25]
Perceived stress (PSS)	20.1 ± 6.9 (1–40)
Burnout (MBI)—emotional exhaustion	17.3 ± 10.8 (0–50)
Burnout (MBI)—depersonalization	5.3 ± 5.4 (0–25)
Burnout (MBI)—personal accomplishment	32.8 ± 9.8 (0–48)
Life satisfaction (SWLS)	26.0 ± 6.1 (5–35)
Life orientation (LOT-R)-optimism	3.8 ± 0.8 (1–5)
Life orientation (LOT-R)-pessimism	2.4 ± 0.8 (1–5)
Self-efficacy (GES)	26.7 ± 5.7 (3–40)
Sense of coherence (SOC)	62.9 ± 10.0 (26–86)
Spiritual engagement (mean ± SD, range)	
Daily spiritual experiences (DSEs)	4.0 ± 0.9 (1–6)
Importance of active religious practices (SpREUK-P Ipt)	61.8 ± 18.3 (0–100)
Importance of gratitude/awe (SpREUK-P Ipt)	70.1 ± 21.6 (0–100)
Importance of passive religious activities (SpREUK-P Ipt)	64.9 ± 21.1 (0–100)
Importance of eastern spiritual practices (SpREUK-P Ipt)	8.8 ± 15.1 (0–78)
Importance of existential activities (SpREUK-P Ipt)	63.9 ± 20.8 (0–100)
Importance of humanistic activities (SpREUK-P Ipt)	69.4 ± 15.5 (25–100)

*The relative proportions were referred to the number of respondents.

**Table 2:** Response rates (%).

Item phrasings	Not at all	Rarely	Occasionally	Fairly often	Regularly	No reply
Item 1: I have the feeling that God is distant from me, regardless of my efforts to draw close to him.	35	38	20	6	1	<1
Item 2: I have the feeling that God has abandoned me completely.	53	29	14	4	<1	<1
Item 3: I experience times of “spiritual dryness.”	5	35	46	10	3	<1
Item 4: I have the feeling that I am “spiritually empty.”	17	40	30	9	3	<1
Item 5: I have the feeling that my prayers go unanswered.	17	38	34	9	1	1
Item 6: I know the feeling of not being able to give any more.	9	37	38	13	3	<1

Marker items when these feelings were experienced						

Item 7: These feelings inspire me all the more to help others.	8	14	21	14	2	41
Item 8: I have found ways to deal with these feelings.	3	6	16	25	8	41
Item 9: After these phases of “spiritual dryness” or “abandonment by God,” I experience a greater spiritual serenity and depth.	5	10	26	16	4	39

**Table 3:** Mean values and reliability analysis of the spiritual dryness scale.

Item phrasings	Mean ± SD [0–4]	Item difficulty index (=0.33)	Corrected item-scale correlation	*α* if item deleted (*α* = .867)	Factor loading
Item 1: I have the feeling that God is distant from me, regardless of my efforts to draw close to him.	1.00 ± 0.94	0.25	.733	.831	.838
Item 2: I have the feeling that God has abandoned me completely.	0.69 ± 0.86	0.17	.681	.842	.793
Item 3: I experience times of “spiritual dryness.”	1.70 ± 0.83	0.43	.680	.842	.792
Item 4: I have the feeling that I am “spiritually empty.”	1.41 ± 0.99	0.35	.685	.841	.791
Item 5: I have the feeling that my prayers go unanswered.	1.39 ± 0.92	0.35	.659	.845	.768
Item 6: I know the feeling of not being able to give any more.	1.64 ± 0.94	0.41	.553	.864	.660

Marker items when these feelings were experienced					

Item 7: These feelings inspire me all the more to help others.	1.82 ± 1.07	0.46	—	—	—
Item 8: I have found ways to deal with these feelings.	2.50 ± 1.03	0.63	—	—	—
Item 9: After these phases of “spiritual dryness” or “abandonment by God,” I experience a greater spiritual serenity and depth.	3.05 ± 1.02	0.76	—	—	—

Extraction of the main components (eigenvalue > 1). Varimax rotation with Kaiser's normalization. One factor explains 60% of variance.

**Table 4:** Correlation analyses.

	Spiritual dryness scale (SDS-5)
Daily spiritual experiences (DSEs)	−.660**
Importance of specific spiritual practices (SpREUK-P Ipt)	
Active religious practices	−.453**
Gratitude/awe	−.399**
Passive religious activities	−.263**
Eastern spiritual practices	−.099
Existential activities	−.124
Humanistic activities	−.203**
Health status	
Burnout—emotional exhaustion (MBI)	.464**
Burnout—depersonalization (MBI)	.450**
Burnout—personal accomplishment (MBI)	.441**
Distress—somatization (BSI)	−.261**
Distress—depression (BSI)	.544**
Distress—anxiety (BSI)	.387**
Personal variables	
Age	−.010
Self-efficacy—sum (GSE)	−.305**
Life orientation—optimism (LOT-R)	−.414**
Life orientation—pessimism (LOT-R)	.336**
Satisfaction with life (SWLS)	−.434**
Sense of coherence—sum score (SOC)	−.483**
Work situation	
Duration of work per week	.057
Size of parish/pastoral unit	−.049
Team size	.081
Work engagement (UWES)	−.438**
Perceived stress (PSS)	.466**

*P < .01 (Pearson).

**Table 5:** SDS-5 as predictor of health related variables and spiritual activities.

SDS as predictor of dependent variables, that is,	*R* ^2^	*β*	*T*	*P*
Depression (BSI)	.30	.544	13.27	<.0001
Perceived stress (PSS)	.22	.466	10.71	<.0001
Emotional exhaustion (MBI)	.20	.450	10.05	<.0001

Life orientation—pessimism (LOT-R)	.11	.336	.729	<.0001
Sense of coherence (SOC)	.23	−.483	−11.33	<.0001
Work engagement (UWES)	.19	−.438	−9.96	<.0001

Daily spiritual experiences (DSEs)	.44	−.660	−18.01	<.0001
Importance of active religious activity (SpREUK-P Ipt)	.21	−.453	−10.41	<.0001

**Table 6:** Predictors of SDS-5 (stepwise regression analysis).

Model 4: *R* ^2^ = .55	*β*	*T*	*P*	Tolerance	VIF
(constant)		13.794	.000		
Daily spiritual experiences (DSEs)	−.471	−11.218	.000	.679	1.473
Depression (BSI)	.235	5.170	.000	.577	1.732
Perceived stress scale (PSS)	.148	3.386	.001	.629	1.589
Importance of active religious activity (SpREUK-P Ipt)	−.083	−2.006	.046	.705	1.418

Variables with lacking influence in this model: burnout, sense of coherence, self-efficacy, optimism, and work engagement.

**Table 7:** SDS-5 mean values in individuals with burnout and stress symptoms.

	SDS-5 (mean ± SD)
Emotional exhaustion (MBI) scores	
<15 (48% low)	5.0 ± 3.0
15–21 (21% moderate)	5.6 ± 3.0
>21 (31% high)	8.4 ± 4.2

*F* value	48.6
*P* value	<.0001

Perceived stress (PSS) scores	
<17 (29% absent)	4.4 ± 2.8
17–23 (43% low)	5.9 ± 3.2
24–28 (16% moderate)	7.5 ± 3.3
>28 (12% high)	9.7 ± 4.4

*F* value	35.5
*P* value	<.0001

Daily spiritual experiences scale (SDES) scores	
<3 (11% low)	11.4 ± 3.9
3–4.9 (72% moderate)	6.1 ± 2.9
≥5 (17% high)	3.1 ± 2.4

*F* value	111.5
*P* value	<.0001
